# Morphological defects in native Japanese fir trees around the Fukushima Daiichi Nuclear Power Plant

**DOI:** 10.1038/srep13232

**Published:** 2015-08-28

**Authors:** Yoshito Watanabe, San’ei Ichikawa, Masahide Kubota, Junko Hoshino, Yoshihisa Kubota, Kouichi Maruyama, Shoichi Fuma, Isao Kawaguchi, Vasyl I. Yoschenko, Satoshi Yoshida

**Affiliations:** 1 Fukushima Project Headquarters, National Institute of Radiological Sciences, Chiba 2638555, Japan; 2 Japan Wildlife Research Centre, Tokyo 1308606, Japan; 3 Tokyo College of Environment, Tokyo 1308606, Japan; 4 Institute of Environmental Radioactivity, Fukushima University, Fukushima 9601296, Japan

## Abstract

After the accident at the Fukushima Daiichi Nuclear Power Plant (F1NPP) in March 2011, much attention has been paid to the biological consequences of the released radionuclides into the surrounding area. We investigated the morphological changes in Japanese fir, a Japanese endemic native conifer, at locations near the F1NPP. Japanese fir populations near the F1NPP showed a significantly increased number of morphological defects, involving deletions of leader shoots of the main axis, compared to a control population far from the F1NPP. The frequency of the defects corresponded to the radioactive contamination levels of the observation sites. A significant increase in deletions of the leader shoots became apparent in those that elongated after the spring of 2012, a year after the accident. These results suggest possibility that the contamination by radionuclides contributed to the morphological defects in Japanese fir trees in the area near the F1NPP.

During the Fukushima Daiichi Nuclear Power Plant (F1NPP) accident that occurred in March 2011, radionuclides that were released into the atmosphere contaminated the surrounding environment^1^,^2^. Since the accident, much attention has been paid to the biological consequences of contamination by radionuclides. To detect the biological changes in the environment, various wild organisms, such as Japanese monkeys^3^, lycaenid butterflies^4^, and gall-forming aphids^5^, inhabiting the surrounding area have been investigated as possible indicator organisms. However, further studies using radiation-responsive indicator organisms help us to reach a consistent conclusion, whether radiological contamination from the F1NPP accident had a biological impact on the environment.

For the purpose of biomonitoring of the radiological contamination, nevertheless, coniferous plants have been demonstrated to be suitable indicator organisms because of their high radiosensitivity, which was revealed decades ago by field examination using gamma irradiation facilities^6^,^7^,^8^,^9^. Radiosensitive damages in conifers were reported after the Chernobyl nuclear accident in 1986, where two local coniferous species, Scots pine (*Pinus sylvestris*) and Norway spruce (*Picea abies*), showed distinct biological damage in the radioactively contaminated areas^10^,^11^,^12^. Under experimental and accidental exposure, morphological changes, particularly in branching of the main axis, were shown to be the most frequently observed radiosensitive responses of coniferous plants^6^,^7^,^8^,^9^,^10^,^11^,^12^.

Coniferous tree species are grown in the area highly radioactive contaminated by the F1NPP accident, where Japanese fir (*Abies firma*) is one of the most common naturally grown species. Different from other coniferous species, young-tree populations of Japanese fir are abundant, because this species has the characteristic ability to sprout even on the shaded forest floor. The short height of young trees enables the easy observation of morphological changes in the whole tree. In addition, the regular annual branching of Japanese fir trees enables determination of the year that any morphological changes occurred through a number of past years (Fig. 1).

In this study, we used the Japanese fir tree as an indicator organism to detect the environmental impact of radiological contamination caused by the F1NPP accident. We examined the morphological changes in annual leader shoots for the past five years within the highly contaminated area around the F1NPP^13^. The investigation was carried out in January 2015 at 3 observation sites (S1, S2, and S3), at different distances from the F1NPP and with different contamination levels (Fig. 2, Table 1). The three observation sites were situated in “Area 3” where it is expected that the residents have difficulties in returning for a long time (Ministry of Economy, Trade and Industry). Fir trees were also examined in a slightly contaminated control site (S4), southwest of the F1NPP.

## Results

Most of the naturally grown Japanese fir trees showed a typical monopodial branching pattern to form a trunk with one main axis (Fig. 3A), whereas some trees showed distinctive morphological defects on the main axis of the trunk (Fig. 3B,C). Independently of the growing site, these defects were characterized by irregular branching at the whorls of the main axis with a distinct deletion of the leader shoot that normally elongates vertically to form the main axis. The space of the deleted leader shoot was filled in by the remaining lateral branches that either extended upwards (Fig. 3B) or retained their horizontal position (Fig. 3C).

The overall frequency of the morphological defects of the main axis varied among observation sites, S1, S2, and S3, but it was significantly higher in each site compared to the control, S4 (chi-square test with df = 1, *p* = 2.1 × 10^−58^, 3.7 × 10^−17^, and 8.1 × 10^−7^, respectively, Bonferroni-corrected; Fig. 4). The frequency corresponded to the ambient dose rate at the observation sites that represented the local levels of radionuclide contamination (S1 > S2 > S3 > S4, Table 1). A high frequency of defects was observed in S1, where 125 out of 128 trees showed branching defects of the main axis.

Branching defects of the main axis were analyzed separately in each annual whorl (Fig. 5). Compared to the whorls of 2010, which had been generated before the F1NPP accident, the frequency of deleted leader shoots was significantly increased in the whorls after 2012 (sites S1 and S3), or those after 2013 (site S2). The frequency peaked in the whorls of 2013 and tended to decrease in the whorls of 2014 in every observation site. The variation patterns in the series of annual whorls were similar among the sites, whereas no annual variation was observed in the control site, S4. These results indicated that the deletion of leader shoots occurred most frequently in the whorls that elongated from terminal winter buds during the growing season of 2012–2013.

Despite the significant increase in the frequency of deleted leader shoots in annual whorls around 2013 in the observation sites S1–S3, the number of lateral branches that elongated from the same whorls did not show annual variation that corresponded to the deletion frequency of leader shoots (Fig. 6). The number of lateral branches was not different among annual whorls even in S1 (one-way ANOVA, *p* = 0.84), in which the frequency of leader shoot deletions varied most intensely compared to the other observation sites (Fig. 5). On the other hand, the number of lateral branches showed significant annual variation in S2, S3, and S4 (one-way ANOVA, *p* = 1.4 × 10^−7^, 6.3 × 10^−3^ and 1.5 × 10^−8^ for S2, S3, and S4, respectively); however, the annual variation patterns were independent from the frequency of leader shoot deletions. In addition, the variation in lateral branch number among the sites did not correspond to the frequency variation of deleted leader shoots. This indicated that the deletion of leader shoots occurred independently of the change in lateral branch number that elongated from the whorls.

Differences in the development of the leader shoots and lateral branches were also observed from a close inspection of the defected whorls. At each site, the deleted leader shoots left no marks among normal lateral branches (Fig. 7A). Similar structures were also observed in the winter buds of 2015 at the top of the main axis, where normal lateral buds with completely deleted apical buds were sometimes observed (Fig. 7B). These observations demonstrated that the deletion of leader shoots probably resulted from the deletion of apical buds at an early stage of their development, independently of the formation of lateral buds.

## Discussion

In this study, significant increases in the morphological defects were shown in Japanese fir populations growing in areas near the F1NPP. The occurrence corresponded to the radioactive contamination level represented by the ambient dose rate in each site, suggesting that the defects could be due to the exposure to ionizing radiation from the radionuclides released after the accident.

On the other hand, deletion of leader shoots was also observed in the control site at a lower frequency, indicating that the defects were not radiation-specific, but universal. The deletion of leader shoots in the control site occurred randomly in the annual whorls and not specifically in a certain year. Moreover, even in the highly contaminated sites, a low frequency of defects was observed before the F1NPP accident in 2011. These results suggested that the defects could also occur independently of radiation exposure.

Similar defects of the main axis have been reported in many coniferous species grown in plantations and involve the separation of trunk into two or more stems of similar size, which is called a forking defect^14^,^15^,^16^,^17^. Forking defects can be caused by breakage of the leader shoot due to an accidental damage, such as bird perching, animal attack, wind damage, and pathogenic disease, or due to environmental stress such as frost^14^. Previous studies have shown that in lodgepole pine (*Pinus contorta*), the forking defects could also be caused by physiological control of apical dominance even in the absence of mechanical damage^14^,^15^. In this study, the observed forking defects in Japanese fir were identical to those in other coniferous tree species.

In relation to radiation effects, deletion of the leader shoots has been reported in Scots pine trees chronically exposed to radiation in a contaminated area close to the Chernobyl nuclear power plant^11^. The trees that showed forking defects with deletion of annual leader shoots eventually formed bushy canopies without a main axis. Another study showed that Scots pine trees in Chernobyl were characterized by the disappearance of a single trunk and replacement with two or more trunks or branches, corresponding to the estimated dose rate during the development of apical buds^12^. Although the defects in pine trees close to the Chernobyl nuclear power plant were not all identical to the defects observed in Japanese fir trees in the area close to the F1NPP, the information seems to support the relationship between the morphological changes in Japanese fir and the chronic exposure to radiation from released radionuclides.

Despite the correlation between the defects in Japanese fir and the radioactive contamination level, there is little biological information to support the contention that the increased frequencies of the morphological changes were due to radiation released after the F1NPP accident. Even though the damage at the early stage of apical bud formation is suggested as the main cause of the deletion of leader shoots, there was an inexplicable 2-year time lag between 2011, the year with the highest radiation dose in the environment, and 2013, the year with the highest frequency of defects. Consequently, processes at the cellular and tissue level involved in the deletion of leader shoots need to be elucidated in relation to the development of lateral and apical buds in coniferous plants.

As described above, there are several factors that are possibly responsible for increased frequencies of the morphological defect observed in Japanese fir populations near the F1NPP, and, at present, there is no decisive evidence that any single factor is causally related to these increased frequencies. However, a positive correlation was observed between ambient dose rates and frequencies of the morphological defect, and these frequencies increased after the F1NPP accident while they were much lower before the accident, suggesting that, of several potential factors, ionizing radiation is most likely to have increased frequencies of the morphological defect. To confirm this contention, dose rates to Japanese fir should be estimated in contaminated fields of Fukushima, and effects of long-term irradiation on this tree should be investigated in irradiation facilities.

## Methods

### Plant and field observations

**Figure 1:**
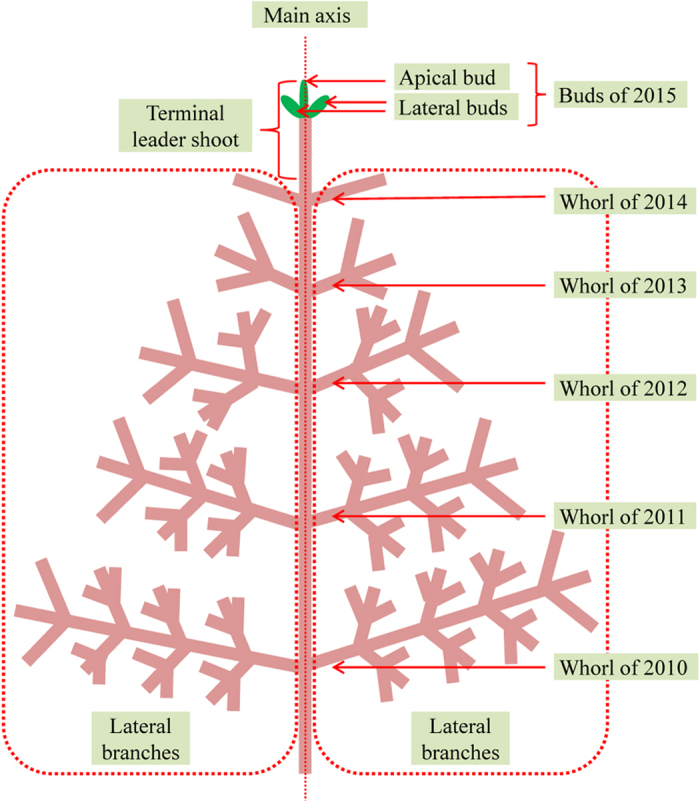
Schematic diagram of Japanese fir tree in January 2015.

**Figure 2:**
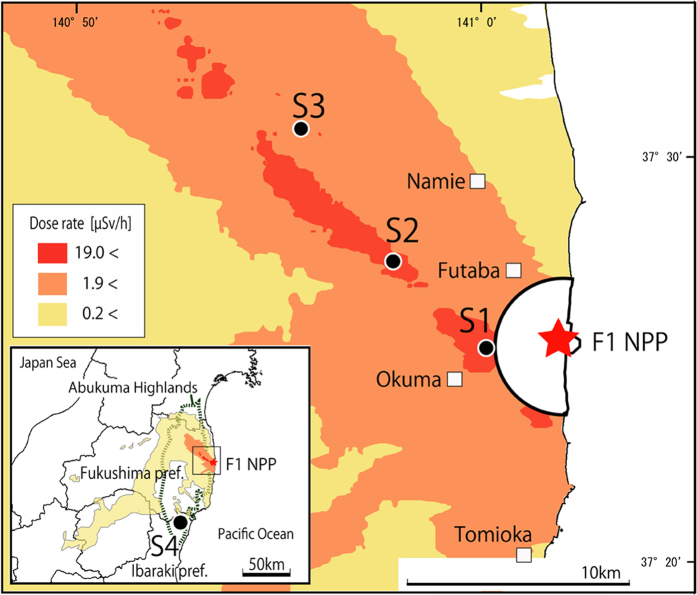
Map showing the observation sites. A red star indicates the location of the Fukushima Daiichi Nuclear Power Plant. The base map was modified from the Digital Japan Portal Web Site (Geospatial Information Authority of Japan; GSI). Airborne monitoring results on 2013.11.19; the “Extension site of distribution map of radiation dose, etc.,/Digital Japan”^13^ was used as the source of the distribution of radiation dose.

**Figure 3:**
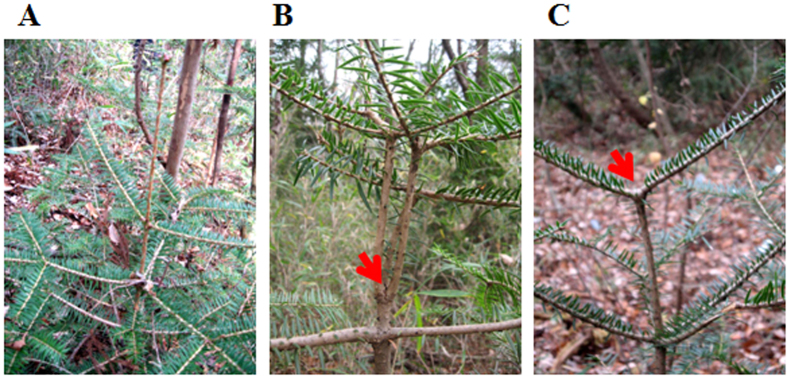
Representative morphological defects in Japanese fir trees. Arrowheads indicate the position of deleted leader shoot. (**A**) normal tree (S3), (**B**) defected tree (vertical forking, S1), (**C**) defected tree (horizontal forking, S2).

**Figure 4:**
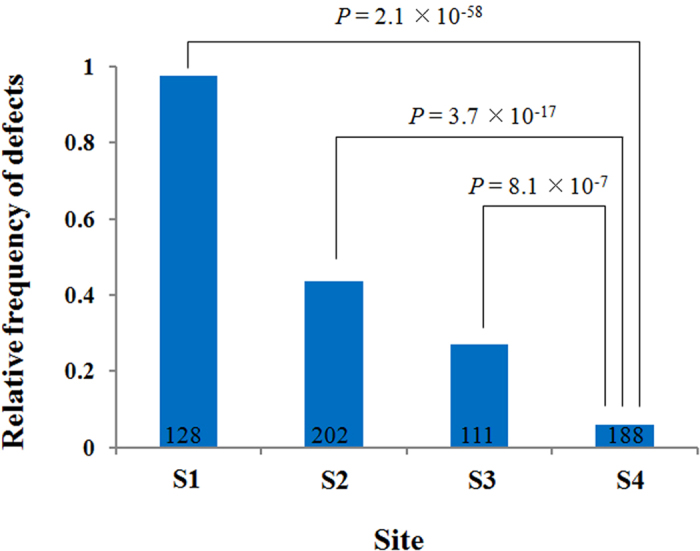
Relative frequency of main axis defects in Japanese fir trees from different sites. The defects were counted by the observation of 5 annual whorls from the top of the trees. Trees with deleted or dead leader shoots in the lower whorls were excluded from observation; therefore the observed tree number for defects (n: indicated in the graph) were different from the total number of observed trees (Table 1). Bonferroni-corrected *p*-values are presented using Chi-square tests with df = 1.

**Figure 5:**
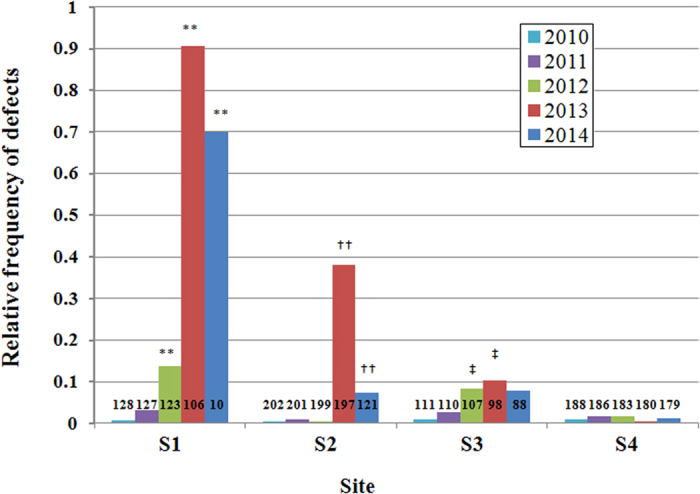
Relative frequency of deleted leader shoot in the annual whorls of the main axis in Japanese fir trees from different sites. The deletions were counted by the observation of 5 annual whorls from the top of the trees. Years indicate the time that the whorl branches sprouted out in the spring (Fig. 1). Trees with deleted or dead leader shoots by previous years were excluded from observation; therefore observed tree number (n: indicated in the graph) decreased in each year. Different letters indicate statistically significant differences compared to the whorls of 2010 in each site (**^, ††, ‡‡^*p* < 0.01, *^,†, ‡^*p* < 0.05, Bonferroni-corrected Fisher’s exact test). *P* < 0.0001 for each of 2012, 2013, and 2014 in S1; *P* < 0.0001 and *P* = 0.0032 for 2013 and 2014, respectively in S2; *P* = 0.0032 and 0.014 for 2012 and 2013, respectively in S3.

**Figure 6:**
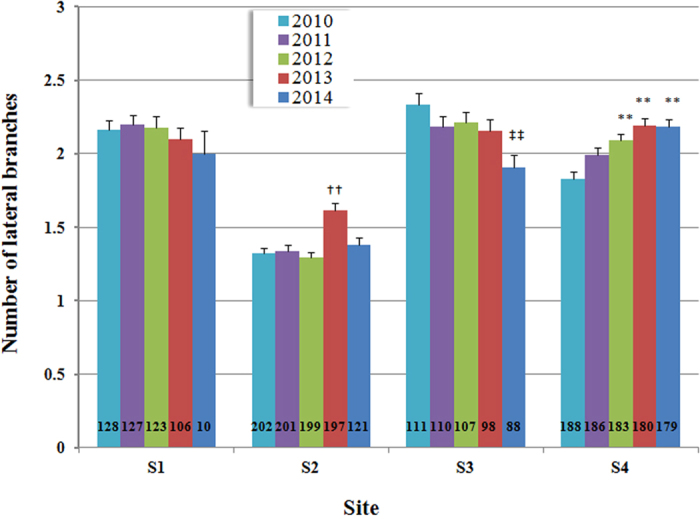
Number of lateral branches from the annual whorls of the main axis in Japanese fir trees from different sites. The lateral branches were counted by the observation of 5 annual whorls from the top of the trees. The data are shown as mean ± SE. Years indicate the time that the whorl branches sprouted out in the spring. Trees with deleted or dead leader shoots by previous years were excluded from observation; therefore observed tree number (n) decreased in each year (n is indicated in the graph). Different letters indicate statistically significant differences compared to the whorls of 2010 in each site (**^, ††, ‡‡^*p* < 0.01, *^,†, ‡^*p* < 0.05, Bonferroni-corrected t test after one-way ANOVA). *P* < 0.0001 for 2013 in S2; *P* = 0.0012 for 2014 in S3; *P* = 0.00013 for 2012, and *P* < 0.0001 for each of 2013 and 2014 in S4.

**Figure 7:**
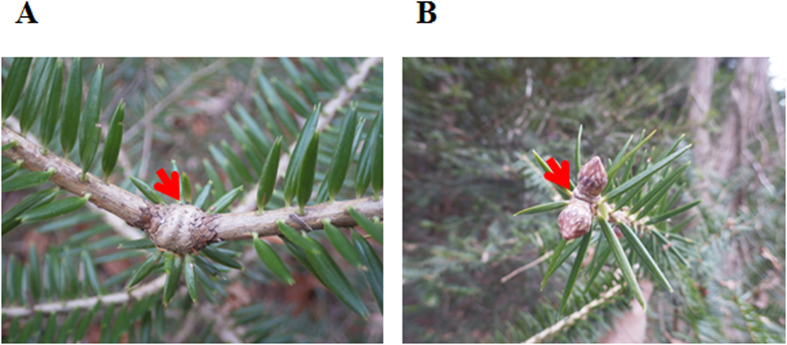
Representative deleted leader shoot and apical bud of main stem in Japanese fir trees. Arrowheads indicate the position of deleted leader shoot. (**A**) annual whorl of 2013 (S1), (**B**) winter buds of 2015 at the top of the main axis (S1).

Samples of Japanese fir were examined in 4 sites (3 observation sites and 1 control site) in the Abukuma region dominated by the Abukuma Highlands and a series of gentle hills leading to a narrow plain along the east coast of Pacific Ocean (Fig. 2, Table 1). Climate conditions in this region are commonly cold with little snow during the winter. The vegetation in the examined sites was mostly mixed forests of Japanese fir trees with other dominant trees such as Japanese red pine (*Pinus densiflora*), and oak (*Quercus serrata*) (Table 1). The investigation of fir trees was performed between January 5 and 9, 2015, approximately 4 years after the F1NPP accident in March 2011. At the time of investigation, fir trees were in their dormant season when each branch has apical and lateral buds at the top (Fig. 1).

All the fir trees between 0.4 m and 5 m in height were observed within 8–12 quadrats (10 × 10 m) placed in each examined site. Five whorls from the top of the main axis were observed. The occurrence of deleted leader shoots and the number of lateral branches were determined in each of annual whorl. The counts and the number in all the quadrats within each site were pooled before analysis.

### Measurement of ambient dose rate

The ambient dose rate was measured at the centre and corners within each quadrat at 1 m above ground level with either an ionization chamber-type survey meter (ICS-323C, Hitachi Aloka Medical, Tokyo) for S1 or a NaI scintillation survey meter (TCS-172, Hitachi Aloka Medical, Tokyo) in S2, S3, and S4. The measurements were averaged within each site to determine the representative value of the ambient dose rate in the site.

### Statistical analysis

Data were analyzed using Excel 2007. Comparisons between the groups were performed using Fisher’s exact test or the Chi-squared test.

## Additional Information

**How to cite this article**: Watanabe, Y. *et al.* Morphological defects in native Japanese fir trees around the Fukushima Daiichi Nuclear Power Plant. *Sci. Rep.* **5**, 13232; doi: 10.1038/srep13232 (2015).

## Figures and Tables

**Table 1:** Observation sites of fir trees in January 2015.

**Site**	**Location**	Direction anddistance fromthe F1NPP*	Date ofexamination	Ambientdose rate(μSv/h)	Vegetation(dominant species)	Forestcanopyheight(m)	Examinedarea (m^2^)	Numberof treesobserved
S1	37°25′N 141°00′E	3.5 km NW	2015/1/6	33.9 ± 5.1	Oak forest (*Quercus serrata*)	18	1100	130
S2	37°26′N 140°59′E	8.5 km WNW	2015/1/8	19.6 ± 1.0	Pine forest (*Pinus densiflora*)	20	1000	203
S3	37°28′N 140°57′E	15 km WNW	2015/1/7	6.85 ± 1.19	Oak forest (*Quercus serrata*)	14	800	111
S4	36°51′N 140°59′E	75 km SSW	2015/1/9	0.13 ± 0.01	Oak forest (*Quercus serrata*)	18	1200	189

^*^F1NPP: Fukushima Daiichi Nuclear Power Plant.
